# Open reduction and internal fixation compared to closed reduction and external fixation in distal radial fractures: A randomized study of 50 patients

**DOI:** 10.3109/17453670903171875

**Published:** 2009-08-01

**Authors:** Antonio Abramo, Philippe Kopylov, Mats Geijer, Magnus Tägil

**Affiliations:** ^1^Hand Unit, Department of Orthopedics, Clinical Sciences, Lund UniversitySweden; ^2^Department of Radiology, Lund University HospitalSweden

## Abstract

**Background and purpose** In unstable distal radial fractures that are impossible to reduce or to maintain in reduced position, the treatment of choice is operation. The type of operation and the choice of implant, however, is a matter of discussion. Our aim was to investigate whether open reduction and internal fixation would produce a better result than traditional external fixation.

**Methods** 50 patients with an unstable or comminute distal radius fracture were randomized to either closed reduction and bridging external fixation, or open reduction and internal fixation using the TriMed system. The primary outcome parameter was grip strength, but the patients were followed for 1 year with objective clinical assessment, subjective outcome using DASH, and radiographic examination.

**Results** At 1 year postoperatively, grip strength was 90% (SD 16) of the uninjured side in the internal fixation group and 78% (17) in the external fixation group. Pronation/supination was 150° (15) in the internal fixation group and 136° (20) in the external fixation group at 1 year. There were no differences in DASH scores or in radiographic parameters. 5 patients in the external fixation group were reoperated due to malunion, as compared to 1 in the internal fixation group. 7 other cases were classified as radiographic malunion: 5 in the external fixation group and 2 in the internal fixation group.

**Interpretation** Internal fixation gave better grip strength and a better range of motion at 1 year, and tended to have less malunions than external fixation. No difference could be found regarding subjective outcome.

## Introduction

Distal radial fractures account for about one-sixth of the fractures seen in the emergency room, with an annual incidence of 26 per 10,000 inhabitants in Sweden (Brogren et al. 2007). Non-operative treatment using plaster cast is chosen in non-displaced fractures and in displaced, but reducible fractures (Handoll and Madhok 2003a). The subject of our study is: fractures that are primarily impossible to reduce or impossible to retain in an acceptable position. These fractures are often considered necessary to operate. The type of operation and the choice of implant is still, however, a matter of discussion; a Cochran report has stated that “randomized trials do not provide robust evidence for most of the decisions necessary in the management of these fractures” (Handoll and Madhok 2003b).

At our department, 2 types of surgical interventions have been used over the last decade for the treatment of distal radius fractures. The TriMed fragment-specific system (Schnall et al. 2006), is used preferably in younger patients whereas external fixation has been used more in older patients, but is still an acceptable option in all age groups. The present randomized study was conducted to compare closed reduction combined with external fixation—which has been or still is the standard operation in many hospitals—to the more complex and more technically demanding open reduction and internal fixation. Our aim was to determine whether a more accurate reduction could be achieved and retained during healing, and whether the outcome—both objective and subjective—could be improved by internal fragment-specific fixation methods, compared to external fixation. The study allowed the best possible operation performed either openly or closed—thus allowing for additional pins, bone substitute, or graft if deemed necessary. As primary outcome, we chose grip strength at 7 weeks and 12 months postoperatively and as secondary outcome we chose the DASH score at the same 2 time points.

## Patients and methods

### Patients

At our department, all patients with a distal radial fracture are treated according to a treatment protocol (Abramo et al. 2008). Non-displaced fractures are treated in a plaster cast for 4–5 weeks. Displaced fractures are reduced and casted. If the fracture after reduction is unstable or even impossible to primarily reduce (for definitions, see Table 1), surgical treatment is suggested to the patient. Patients with fractures in the AO groups A1–3 and C1–3 were eligible for the study. These patients were invited to participate in a randomized study comparing open and closed treatment. The study was approved by the local ethics committee (no. Lu 45/02).

**Table 1.** The inclusion and exclusion criteria for the study

*Inclusion criteria*
Age 18–65
Frykman type I–VIII fracture impossible to reduce or retain in an acceptable position in cast after closed reduction
Injury less than 10 days previously
Incongruence in RC-or DRU-joint and/or axial compression > 2 mm, and/or dorsal angulation > 20°
Patient had received oral and written information and signed an informed consent
*Exclusion criteria*
Previous ipsilateral fracture
Volarly displaced fracture
Fracture in the contralateral side, or other fracture in need of treatment
Open fracture
Ongoing radiotherapy or chemotherapy
Metabolic disease affecting the bone
Medication affecting the bone
Dementia, psychiatric disorder, or alcohol abuse

Between May 2002 and December 2005, 50 patients (36 women) with a mean age of 48 (20–65) years with unstable fractures fulfilled the inclusion criteria (Table 1). Most patients with a distal radius fracture were older than 65 years and were not eligible for the study. Patients with a redislocated fracture were also not eligible for the study. Thus, only younger patients with an unstable fracture who were in need of an acute operation were recruited, thus explaining the relatively long recruitment time.

The patients gave their written and informed consent, and were included and randomized to either open reduction and internal fixation (O), or closed reduction and external fixation (C). 38 patients considered themselves to be healthy, 5 had cardiovascular diseases such as hypertension or atrial fibrillation, 1 had diabetes mellitus, 1 had epilepsy, 1 had hypothyroidism, 1 had well-controlled depressive problems, and 3 had asthma.

### Randomization

Randomization was prepared in blocks of 6 containing equal numbers of C and O patients, and the patients were stratified into 2 age groups. The older group was considered to be more osteoporotic and consisted of men of 60 years of age and above, and women of 50 years of age and above. The younger group of women less than 50 years old and men less than 60 years old were considered to be less osteoporotic. The aim was to recruit at least 24 patients (4 blocks) in each age group and the sealed envelopes were opened on the day of surgery, immediately before the operation. Randomization would stop when 4 blocks (24 patients) in each group had been randomized. 26 patients were randomized to the O treatment and 24 to the C treament.

### Evaluation

All patients were followed for 1 year with visits at 2, 5, and 7 weeks and 3, 6, and 12 months postoperatively. The grip strength at 7 weeks and at 12 months was chosen as the primary outcome and the DASH score at the same time points was chosen as the secondary outcome. Reoperations for either a malunion or a redislocation of the fracture were considered to be endpoints and patients were excluded thereafter. Complications were registered by a hand surgeon at each visit. Complications were divided into (1) major complications, defined as those that were expected to have an effect on the final outcome, (2) moderate complications, defined as those that were not expected to have an effect on the final outcome but would need further interventions, and (3) minor complications, defined as temporary and self-healing. Grip strength (JAMAR), range of motion (goniometer), and sensibility in all fingers (Weber 2PD) were recorded by a physiotherapist at all visits. Lateral and AP radiographs were taken at injury, directly postoperatively, at 2 and 5 weeks, and at 3, 6, and 12 months postoperatively. All radiographs were classified by a radiologist (MG) according to the Frykman and AO classifications. The radiographic result after healing was evaluated according to the same criteria as used for the definition of the primary instability (Table 1). Subjective outcome was evaluated using the DASH score, which is a self-administered questionnaire developed by the AAOS and the Institute for Work and Health in Canada (Hudak et al. 1996). The questionnaire consists of 30 questions evaluating physical activities, severity of symptoms, and the effect of the injury on social activities. A score is calculated and converted to a scale from 0 to 100 with a score of 100 expressing the largest degree of disability. A Swedish version was used, which has been validated for general use in upper extremity disorders (Atroshi et al. 2000). At inclusion, the patients were asked to fill out the DASH questionnaire relating to their pre-injury status and then again at 7 weeks, 3 months, and 1 year postoperatively.

One patient in the O group moved to another part of the country and declined further visits after 7 weeks, when she was back to work and with full function. 1 patient in the O group failed to return the DASH form at 7 weeks, and another in the same group failed to return the form at 3 months. 1 patient in the O group failed to appear at the physical examination at 6 months. 2 patients in the C group failed to appear at the 12-month visit, but returned their completed DASH forms.

### Operative technique

**Figure 1.**
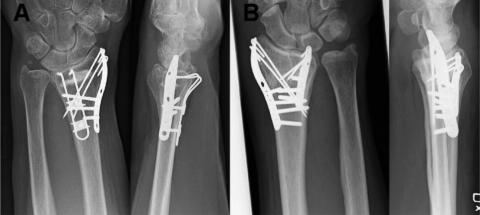
AP and lateral radiographs in two cases of distal radial fracture operated with the TriMed system. A. This patient was operated using a radial pin-plate and a volar buttress pin. Additional stability was achieved using Norian SRS bone substitute. B. In intraarticular fractures with an ulnar fragment, an ulnar pin-plate could be combined with the radial pin-plate.

The patients were operated by 1 of 4 senior hand surgeons. The participating surgeons agreed to aim for the best possible stabilization in each patient with each technique, including the use of additional K-wires, bone graft, or bone substitute. The fragment-specific wrist fixation system TriMed (Konrath and Bahler 2002) was used for internal fixation. The system consists of a combination of pins, plates, and screws (Figure 1). Volar fixed-angle plates were not available at the start of the study and were not used.

*Open reduction and internal fixation (O).* Ordinarily, 2 incisions were made through the first and fourth extensor compartments. The fracture was reduced and 2 pins were introduced at the tip of the radial styloid, obliquely and in a proximal direction—leaving the radial cortex ulnarly and proximally. A stabilizing pin-plate was threaded onto the styloid pins and the plate was secured to the radial side of the radius by 3–5 screws. Through the dorsal incision, a buttress pin and/or an ulnar pin-plate was introduced for dorsal stability. At the surgeon's discretion, Norian SRS (Kopylov 2001) (Synthes GmbH, Switzerland) was used in the void to add stability (2 patients). Postoperatively, the patients were treated with a forearm plaster cast for 2 weeks and thereafter active mobilization was started under the supervision of a physiotherapist.

**Figure 2.**
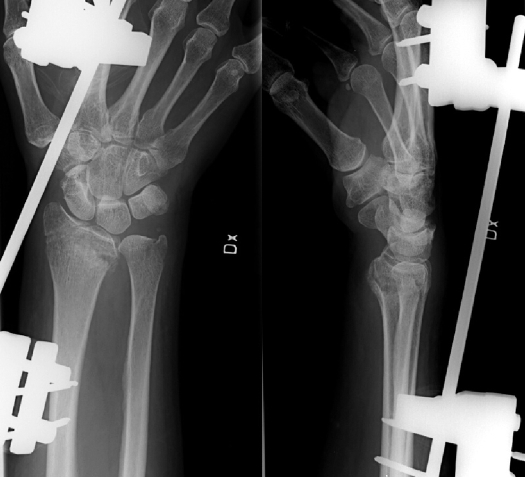
AP and lateral radiographs of a patient operated using closed reduction and external fixation.

*Closed reduction and external fixation (C)*. The external fixator used for the first 20 patients was the Hoffman type-1 bridging external fixator (Stryker, Hopkinton, MA), which was changed to the Radio Lucent Wrist Fixator (Orthofix Srl, Bussolengo, Italy) by the start of 2005 and used in the last 4 patients. Pins were inserted into the second metacarpal and into the radius proximally to the fracture. Clamps were attached to the pins and the fracture was reduced and fixated with a steel rod between the clamps (Figure 2). In comminuted fractures with a bone defect and when additional stability was desired, K-wires were inserted percutaneously. A bone graft substitute (Norian SRS), also inserted percutaneously, was used at the surgeons' discretion (2 patients). The fixator was usually removed after 5–6 weeks and thereafter active mobilization was started under the supervision of a physiotherapist. There was no restriction regarding pronation or supination during the fixation time in either of the groups.

### Statistics

Based on the results of a previous study comparing external fixation with closed treatment using a bone substitute (Kopylov et al. 1999), grip strength was chosen as the primary outcome and a power analysis was performed. 20 patients were needed in each group to show a 10% difference in grip strength with a power of 85% in a two-sided test at the 5% significance level. Fisher's exact test was used for categorized outcomes and Mann-Whitney U test for ordinal outcomes. Student's t-test was used for continuous data such as radiographic measurements. Spearman correlation coefficient was used to calculate correlations between objective and radiographic parameters. SPSS software version 14.0 was used. Bonferroni correction was used for repeated measures of objective parameters at 7 weeks and at 12 months of follow-up.

## Results

Age, sex, injured side, type of work, category of fracture, radiographic findings, and type of injury were equally distributed between the groups (Tables 2 and 3; see supplementary data). Most patients had intraarticular fractures, either in the radiocarpal joint or in the distal radioulnar joint or both, and only 8 patients had extraarticular fractures. There were 4 AO type-A fractures in each group, and 20 type-C fractures in the C group and 22 in the O group.

The operations were performed at a mean time of 3.6 (1–9) days after injury. In 7 patients in the C group, the fracture was augmented with K-wires. Norian SRS was used in 2 patients in each group. Postoperatively, the patients in the open group were treated in a forearm plaster cast for 14 (6–20) days, and the patients in the closed group wore the fixator for 36 (33–41) days. There were no peroperative complications.

### Objective outcome (Figure 3)

At 7 weeks postoperatively, the primary outcome parameter, mean grip strength, was significantly higher in the O group than in the C group (47% of the uninjured side and 34% of the uninjured side, respectively) (p = 0.01). Also, the mean range of motion in forearm rotation was significantly greater in the O group than in the C group (129° and 104°, respectively) (p = 0.006). No statistically significant differences were found regarding extension/flexion (88° and 74°, respectively) (p = 0.09) or radial/ulnar deviation (48° and 41°) (p = 0.2) at the early follow-up. At the final follow-up 1 year postoperatively, a statistically significant difference was still found between the O and C groups both regarding the primary outcome parameter grip strength (90% and 78%, respectively) (p = 0.03) and also forearm rotation (149° and 136°, respectively) (p = 0.03). In both groups, range of movement in extension/flexion was 121° and in radial/ulnar deviation it was 60°.

**Figure 3.**
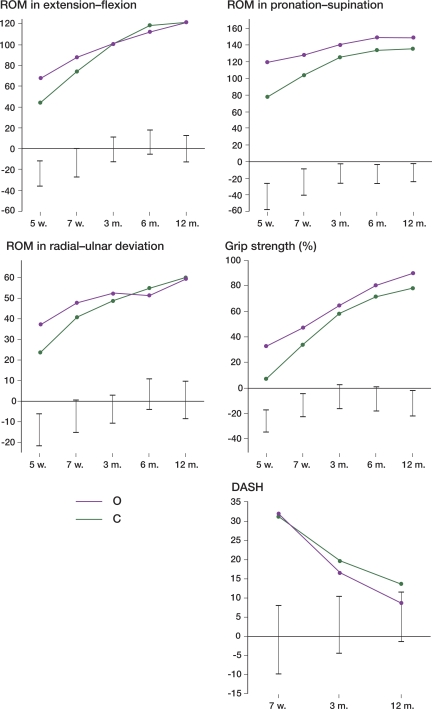
Objective and subjective outcome during the follow-up at 5 and 7 weeks and at 3, 6, and 12 months postoperatively showing range of motion (ROM) in extension/flexion (A), ROM in forearm rotation (B), ROM in radial/ulnar deviation (C), grip strength as a percentage of the opposite side (D), and DASH score (E). Lines represent mean range of motion (degrees) for grip strength (percentage of the opposite side) and DASH score. Error bars represent the 95% confidence interval of the difference between groups.

### Subjective outcome (Figure 3)

The secondary outcome parameter, mean DASH score, was 3 (0–45) before the injury (Table 4) as reported by the patients. 41/48 had a score of 1 or less before injury. 3 patients had a pre-injury DASH score higher than 20, 2 of them due to CMC 1 osteoarthritis, and 1 due to shoulder impingement. The results of the postoperative DASH questionnaires showed no statistically significant differences between the groups at any time after surgery (i.e. 7 weeks, 3 months, or 1 year postoperatively). The DASH scores for the extraarticular fractures were better than the intraarticular scores 3 months postoperatively (median 6.8 vs.17; p = 0.01), but no statistically significant difference was found at 1 year.

**Table 4.** Pre- and postoperative DASH scores

	C		O		
	Mean (SD)	Median (range)	Mean (SD)	Median (range)	p-value **^a^**
Pre-injury	3.6 (10)	0 (0–44)	2.4 (6.2)	0 (0–26)	0.9
7 weeks	31 (16)	29 (5–57)	32 (14)	28 (7–63)	0.5
3 months	20 (14)	14 (0.8–51)	17 (11)	14 (0–43)	0.6
12 months	14 (13)	6.4 (0–35)	8.7 (8.9)	5 (0–31)	0.2

**^a^** Mann-Whitney U test.

### Complications

50 postoperative complications occurred in 34 patients (Table 5). 1 patient in the O group had a postoperative swelling of the hand and fingers, which led to hospitalization for 2 days. Another patient in the same group had a small, incomplete longitudinal fracture proximal to the initial fracture. This was left untreated, and it healed without complications. 2 patients—both in the external fixation (C) group—had early dislocation of the fracture, resulting in both radial compression and angulation requiring surgical correction. 1 patient was reoperated after 2 weeks and the other was reoperated 6 months postoperatively at another hospital. These 2 patients were then excluded from the study analyses.

**Table 5.** Complications by group and severity

Complications	C	O	Total	p-value **^e^**
Minor **^a^**				
Postoperative CTS	3	1	4	
Skin adherences	4		4	
Mb de Quervain		1	1	
Radial neurapraxia	2	10	12	
Adherent tendon		1	1	
Prolonged postoperative pain	4		4	
Pin-tract infection	2		2	
	15	13	28	0.4
Moderate **^b^**				
Postoperative CTS	4		4	
Skin adherences	1		1	
Radial neurapraxia		1	1	
Adherent tendon		1	1	
APL dislocation		1	1	
Radial pin irritation		3	3	
Pin-tract infection	1		1	
	6	6	12	1
Major **^c^**				
Fractured metacarpal	1		1	
Symptomatic malunion **^d^**	5	1	6	
CRPS	2	1	3	
	8	2	10	0.04

**^a^** Minor complications: transient problems with no need for intervention.

**^b^** Moderate complications: complication with a need for further intervention such as surgery or antibiotic treatment, but not affecting the final outcome.

**^c^** Major complications: severe complications influencing the final outcome and in need of surgical or other intervention.

**^d^** Malunions requiring corrective osteotomy.

**^e^** Fisher's exact test.

Most complications in both groups were minor, such as transient carpal tunnel syndrome (CTS) not requiring surgery, skin adhesions, tendonitis not requiring surgery, transient radial neurapraxia, excessive postoperative pain, and superficial infections not requiring antibiotics. The most common minor complication in the O group was radial nerve symptoms, due to the surgical approach through the first extensor compartment for the radial pin-plate. In all cases but 1, the nerve symptoms were transient and had resolved at the final follow-up at 1 year. 1 patient had the plate removed.

Moderate complications requiring secondary interventions but not affecting the final outcome were equally common in both groups. Major complications, which may influence the final outcome, such as malunions requiring additional surgery, splinting, or reflex sympathetic dystrophy, were more common in the C group (Table 5). In the symptomatic malunions leading to a secondary procedure, 1 patient in the C group had a radiocarpal intraarticular malunion and 5 others had extraarticular malunions with shortening and/or angulation of the radius. 5 of these patients were operated with a radial osteotomy, 2 of them also with ulnar shortening. 1 patient in the C group was reoperated with the TriMed, 2 weeks after the primary operation. In addition to the malunions requiring corrective osteotomy, there were 7 other cases of radiographic malunion not in need of further surgery but with an incongruence in either the distal radioulnar joint or in the radiocarpal joint. These malunions are described below in the radiography section. The total number of malunions—those requiring corrective osteotomy and/or radiographic malunions—was 10 in the C group and 3 in the O group.

### Sick leave

Patients with moderate-to-heavy manual work had more days at home from work in the C group than in the O group (Table 6). For patients with desk work, there was no statistically significant difference.

**Table 6.** Days away from work

		C			O		
	n	Mean (SD)	Median (quartile)	n	Mean (SD)	Median (quartile)	p-value **^a^**
Desk work	9	24 (24)	16 (10–33)	6	35 (36)	27 (4–61)	0.4
Manual work	10	89 (35)	87 (76–116)	16	66 (28)	65 (48–82)	0.04
All patients	19	58 (45)	74 (14–88)	22	58 (33)	61 (34–77)	1

**^a^** Mann-Whitney U test.

### Radiology

The fracture types, as classified by the Frykman and by the AO classification, were symmetrically distributed between the groups (Table 3; see supplementary data). As 8 patients in the C group and 10 in the O group underwent closed reduction at the ER prior to the first radiograph, preoperative radiographic measurements could not be done. There were no differences between the groups in mean postoperative radial inclination, dorsal angulation, radial compression, and incongruence in the radiocarpal and the distal radioulnar joint at any time postoperatively (Table 7; see supplementary data). In addition to the 6 malunions requiring corrective osteotomy, there were 7 cases of radiographic loss of correction, 5 in the C group and 2 in the O group. In the C group, 2 cases had intraarticular malunions with intraarticular steps of 2.2 mm and 2.4 mm, 2 cases had ulnar variances of 4.3 mm and 7.9 mm, and 1 case had both a dorsal angulation of 21˚ and an ulnar variance of 4.3 mm. 2 radiographic malunions were seen in the O group, 1 with an articular step of 3.3 mm and 1 with an ulnar variance of 6 mm.

## Discussion

In contrast to many other fractures, there are have been a number of randomized studies on treatment of distal radial fractures. However, no clear conclusions can be drawn from meta-analyses of all randomized radial fracture studies as summarized in the Cochrane report (Handoll and Madhok 2003b) where 48 randomized trials and 25 different treatment options were compared in 3,371 patients. Also, in a major meta-analysis (Margaliot et al. 2005) of 46 non-randomized studies with either external or internal fixation in 1,519 patients, no clear conclusion could be drawn. Finally, in addition to the lack of consensus regarding the older established methods, no randomized or high-quality prospective non-randomized studies have been carried out yet for the newest concepts. We believe that these new concepts, such as the TriMed system used in the present study or the increasingly popular volar angle-stable plates, improve the treatment of unstable distal radial fractures.

To our knowledge, 4 randomized studies have compared open reduction and internal fixation to closed or indirect reduction. In a recent study by Leung et al. (2008), a better result was found for internal fixation with AO plates either dorsally or volarly compared to bridging external fixation with augmentation with Kirschner wires at the surgeon's discretion. The other 3 studies have reported either an absence of significant differences or a better functional outcome for external fixation (Kapoor et al. 2000, Grewal et al. 2005, Kreder et al. 2005). Grewal and co-workers (2005) also found a higher complication rate for internal fixation with a dorsal plate than for external fixation. Kapoor and co-workers (2000) concluded that open reduction and internal fixation provide the best articular anatomy in highly comminuted fractures, although the best outcome was achieved with the external fixator. Grewal et al. (2005) compared internal fixation using the dorsal Pi-plate with mini-open reduction and external fixation, and found a higher complication rate for the Pi-plate. A better grip strength was found in the mini-open group but there were no significant differences in ROM or DASH. Kreder et al. (2005) randomized 179 patients between either a mini-open indirect reduction and K-wires/screws or a full arthrotomy with internal fixation. A better result was found for the indirect group, but a high rate of crossovers from the indirect group to the open group at the time of surgery was reported and many patients were lost to follow-up.

Higher rates of infection and hardware failure have been reported in patients treated with external fixation and higher rates of tendon complications with internal fixation (Margaliot et al. 2005). Thus, in the literature as well as in our study, the patterns of complications differ between the methods and might help the orthopedic surgeon to decide whether to use external or internal fixation. We found a high rate of complications, but most were minor and transient. In the external fixation group, the rate of major complications such as redislocation requiring reoperation or complex regional pain syndrome was higher. Other studies have reported complication rates of 20% and 85% with external fixation (Anderson et al. 2004, Capo et al. 2006), most complications being minor.

The malunion rate is an important outcome variable when evaluating different surgical treatments, and should be included in the overall decision. In our study, 5 cases in the external fixation group and 1 case in the internal fixation group had loss of reduction and malunions requiring further surgery. 5 other patients in the C group and 2 in the O group had radiographic malunion only. The malunion rate found by McQueen (1998), comparing non-bridging external fixation to bridging external fixation, was similar to ours: 14 in the 30 patients treated with bridging external fixator.

Regarding grip strength, which was the primary outcome in the power analysis, the group that was operated with internal fixation had a better result, maybe less surprising, at 7 weeks, but more important also at 12 months. Also, regarding forearm rotation, the results were better in the internal fixation (O) group at all follow-up visits. The absolute values of grip strength and range of motion in the present study were similar to those in other studies, both in the C group (McQueen et al. 1996, Harley et al. 2004, Wright et al. 2005, Atroshi et al. 2006) and in the O group, and in the latter case both comparing to the TriMed system (Benson et al. 2006, Schnall et al. 2006) or to the latest fixation trends of angle-stable volar plating (Musgrave and Idler 2005, Wright et al. 2005).

There may be different explanations for the increased range of motion and grip strength in the internal fixation group after 1 year of follow-up. The fractures in the O group might be better aligned at surgery and/or a better reduction may be maintained during the healing, leading to a better congruency of the joint. In the O group rehabilitation starts 3 weeks earlier, which could explain the early difference between the groups, both regarding range of motion and grip strength, as found in previous studies (Kopylov et al. 1999). However, in the present study, this effect persisted throughout the whole of the first year. Also, regarding the subjective outcome there was a tendency for there to be a better outcome in the O group.

The median DASH values in our series (9 in the O group and 14 in the C group) are similar to the results in other studies reporting DASH scores, around 16 for the volar plate (Musgrave and Idler 2005, Wright et al. 2005), between 9 and 17 for the TriMed system (Konrath and Bahler 2002, Benson et al. 2006, Gerostathopoulos et al. 2007), and between 7 and 17 for external fixation (McQueen et al. 1996, Harley et al. 2004, Wright et al. 2005, Atroshi et al. 2006). This subjective outcome in both groups must be considered favorable, bearing in mind that in our study internal and external fixation was compared in the most unstable distal radial fractures.

In this group of patients with primarily unstable fractures, there is no acceptable alternative to surgery. The two methods we compared will both give a good result with good DASH values, good grip strength, and good range of motion after a year. Overall, considering the subjective and objective results as well as the rate of major complications and the sick-leave, we believe that internal fixation gives a superior result and in our opinion it would be the method of choice; however, results for the external fixator are still acceptable. Which method to use to internally stabilize the fracture is still a matter for discussion and should be the subject of future randomized studies. With smaller and smaller differences between the 2 methods, better and more sensitive subjective outcome instruments will be required if the number of patients needed to show a difference is to be kept within reasonable numbers.
